# A Nomogram for Predicting the Pathological Response of Axillary Lymph Node Metastasis in Breast Cancer Patients

**DOI:** 10.1038/srep32585

**Published:** 2016-08-31

**Authors:** Xi Jin, Yi-Zhou Jiang, Sheng Chen, Zhi-Ming Shao, Gen-Hong Di

**Affiliations:** 1 Department of Breast Surgery, Fudan University Shanghai Cancer Center; Department of Oncology, Shanghai Medical College, Fudan University, Shanghai 200032, China

## Abstract

The value of sentinel lymph node biopsy (SLNB) in post-neoadjuvant chemotherapy (NCT) patients is still controversial. We aimed to identify predictors and construct a nomogram for predicting the pathologically complete response (pCR) of axillary lymph nodes (ALNs) after NCT in node positive breast cancer patients. In total, 426 patients with pathologically proven ALN metastasis before NCT were enrolled, randomized 1:1 and divided into a training set and a validation set. We developed a nomogram based on independent predictors for ALN pCR identified by multivariate logistic regression as well as clinical significant predictors. The multivariate logistic regression analysis showed that hormone receptor (HR) status, human epidermal growth factor 2 (HER2) status and Ki67 index were independent predictors. The nomogram was thereby constructed by those independent predictors as well as tumor size and NCT regimens. The areas under the receiver operating characteristic curve of the training set and the validation set were 0.804 and 0.749, respectively. We constructed a nomogram for predicting ALN pCR in patients who received NCT. Our nomogram can improve risk stratification, accurately predict post-NCT ALN status and avoid unnecessary ALN dissection.

Breast cancer has been reported to be the most common malignant tumor and the second leading cause of cancer death among women in America. The incidence of breast cancer is increasing^1^. The utilization of neoadjuvant chemotherapy (NCT) can reduce tumor burden, help to increase the curative intervention as well as the breast conservation rate, and provide the opportunity to assess the response to treatment^2^.

Mastectomy together with routine axillary lymph node (ALN) dissection used to be the standard type of surgery for breast cancer patients^3^. However, the dissection of the ALN can cause several physical and psychological morbidities, including swelling, weakness and stiffness of the upper limb, pain and numbness in the axillary, and movement disorders of the shoulder girdle as well as damage to social functions^4^,^5^. Sentinel lymph node biopsy (SLNB) is the alternative treatment to ALN dissection. Several randomized clinical trials showed that SLNB was associated with similar therapeutic effects as well as reduced arm morbidities (including edema and nerve injury) and a better quality of life compared with standard ALN dissection^6^,^7^.

The use of SLNB in patients after receiving NCT is still in doubt because chemotherapy can cause fibrosis of the lymphatic duct leading to the sentinel lymph nodes, thus interfering with the accuracy of SLNB^8^. Previous research demonstrated that the false negative rate (FNR) of biopsy proven node positive breast cancer patients who received SLNB post-NCT ranged from 5.1% to 25%^9^,^10^,^11^,^12^. Therefore, performing SLNB post-NCT is controversial. However, none of the previous studies focused on screening node positive breast cancer patients who had a high probability of achieving ALN pathological complete response (pCR) or increasing the accuracy of SLNB post-NCT.

Our current research is intended to identify possible predictors and construct a nomogram for predicting pCR of ALN post-NCT among node positive breast cancer patients, which will increase the accuracy of SLNB post-NCT. In combination with SLNB and nomogram prediction, it may be possible for patients with a high probability of ALN pCR to avoid ALN dissection.

## Methods

### Patient population

Relevant clinical information (age, menopausal status, tumor size and NCT cycles), ALN status, core needle biopsy (CNB) samples from primary tumors and surgical specimens were collected from 1244 consecutive patients who received NCT as well as standard surgery at the Fudan University Shanghai Cancer Center (FUSCC) between January 1, 2003 and April 30, 2015.

The eligibility criteria included: 1) diagnosed with primary breast cancer, 2) received NCT, and 3) had pre-NCT axillary nodal disease confirmed by pathology. Patients with missing data, uncertain pre-NCT axillary nodal status or negative nodal disease confirmed by pathology, or with distant metastatic disease were excluded. The eligible patients were randomized 1:1 and divided into a training set (nomogram construction) and a validation set (nomogram validation).

### Pathology

The ALN status before NCT was evaluated by fine needle biopsy. The hormone receptor (HR) status, the human epithelial growth factor receptor 2 (HER2) status and the Ki67 index were evaluated by immunohistochemical (IHC) and fluorescence *in situ* hybridization (FISH) analyses, which were performed on formalin-fixed, paraffin-embedded tissue sections by the pathology department of FUSCC using standard protocols for specimens from CNB. The cut-off value for estrogen receptor (ER) positivity and progesterone receptor (PR) positivity was set at 1%. The absence of both ER and PR was defined as HR negative; the presence of either was defined as HR positive. HER2 positive was defined as 3(+) according to IHC analysis or amplification confirmed by FISH; lower scores were defined as HER2 negative. The Ki67 expression was divided into two groups: Ki67 > 20% and Ki67 ≤ 20%^13^. Each specimen was examined independently by two experienced pathologists at FUSCC.

The pCR of the ALN was defined as the complete disappearance of invasive carcinoma of the regional lymph nodes after the patients received mastectomy with axillary lymph node dissection^14^,^15^.

### Treatment

The patients in our cohort received an NCT regimen consisting of epirubicin + paclitaxel contained (E + P, include cyclophosphamide + epirubicin + 5-fluorouracil followed by paclitaxel as well as docetaxel + epirubicin) and PC (paclitaxel and carboplatin or paclitaxel and cisplatin) for a median of 4 cycles (range, 1–8 cycles). After 2007, trastuzumab (Herceptin, H) was recommended for HER2 positive patients combined with chemotherapy as the neoadjuvant therapy regimen.

### Nomogram construction and validation

To develop a well-calibrated nomogram for predicting the probability of pCR of the ALN, we performed univariate as well as multivariate logistic regression analyses to screen the fit predictors^16^. The correlation between clinicopathological variables and ALN pCR of overall population, training set and validation set were Variables that were statistically significant (P < 0.05) in the univariate logistic analysis in the training set were included in the multivariate logistic regression analysis, which was performed to screen independent predictors for ALN pCR. Independent predictors (P < 0.05 in the multivariate logistic regression analysis) as well as clinical significant predictors were included in the nomogram construction. The goodness of fit of the model was assessed by the Hosmer and Lemeshow test, and P > 0.05 indicated a good fit^17^. The odds ratios (ORs) and 95% confidence intervals (CIs) were also calculated.

### Evaluating nomogram performance

The nomogram was validated internally in the training set and externally in the validation set. The internal validation was performed by a calibration method and the area under the receiver operating characteristic (ROC) curve (AUC). The external validation was performed by calculating the AUC. The calibration plot with bootstrapping was used to illustrate the association between the actual probability and the predicted probability^18^. The AUC ranged from 0 to 1, with 1 indicating perfect concordance, 0.5 indicating no better than chance, and 0 indicating discordance. Statistical differences between different AUCs were investigated by the DeLong method^19^. The diagnostic odds ratio was calculated to further evaluate the performance of the nomogram^20^. The diagnostic odds ratio ranges from 0 to infinity (higher values indicating better performance of a discriminatory test). A value of 1 means that a test does not discriminate between patients with the disorder and those without it^20^.

All reported P values are two-sided. The statistical analysis was carried out using SPSS (version 20.0; SPSS Company, Chicago, IL) and R software version 3.13 (http://www.r-project.org). The R package with the rms, pROC, Hmisc, and ggplot2 (available at URL: http://cran.r-project.org/web/packages/) was used (last accessed on March 9, 2015). All relevant R code were shown in Supplementary material.

### Ethical approval

All the procedures followed were in accordance with the Helsinki Declaration (1964, amended in 1975, 1983, 1989, 1996 and 2000) of the World Medical Association. This study was approved by the Ethics Committee of FUSCC, and each participant signed an informed consent document.

## Results

### Patient characteristics

**Figure 1:**
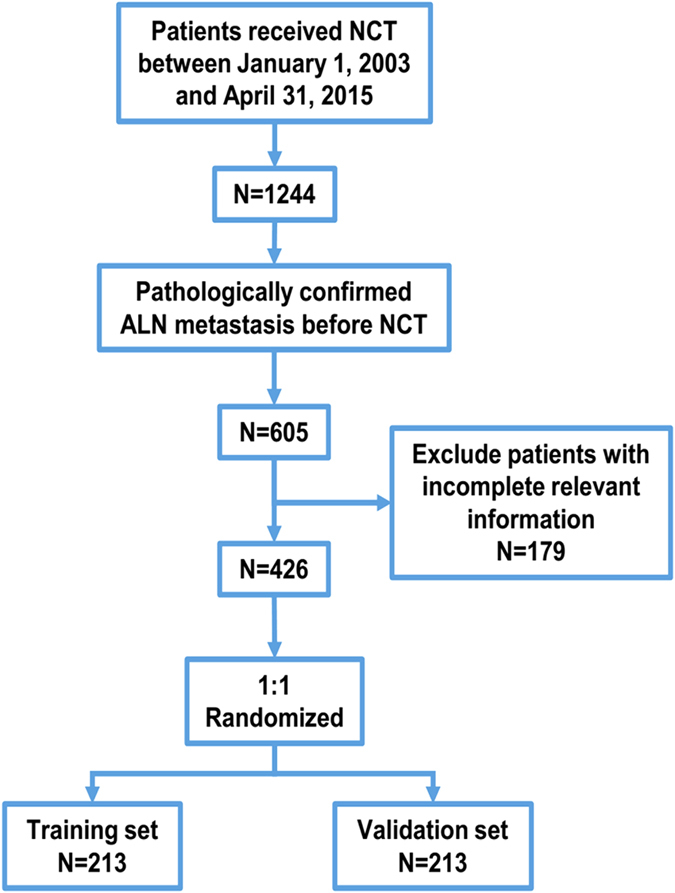
Flow diagram of the study design. A total of 426 patients with axillary lymph node (ALN) metastasis pathologically confirmed by fine needle biopsy who received neoadjuvant chemotherapy (NCT) were enrolled in this study.

Among 1244 patients, 605 of them were pathologically confirmed ALN metastasis before NCT. After excluding 179 patients with incomplete relevant information, 426 eligible patients were included in the study (Fig. 1). The 426 patients were randomized 1:1 and divided into a training set (N = 213) and a validation set (N = 213). The clinicopathological characteristics and the univariate logistic regression analysis of the total population, the training set and the validation set are shown in Table 1. Of the 426 patients, 128 (30.0%) showed ALN pCR after receiving NCT. Younger patients (≤40y, 30.7%) had an ALN pCR rate similar to that of elder patients (>40y, 29.9%, P = 0.897). Similar results were observed among pre-menopausal patients and post-menopausal patients (P = 0.849). Patients with a tumor size of T1 had a higher probability of showing axillary pCR than those with sizes T2, T3 or T4 (OR: 1 vs 0.391 [95% CI: 0.193–0.793], 0.195 [95% CI: 0.082–0.465] and 0.304 [95% CI: 0.138–0.670]). The HR negative patients were more likely to show axillary pCR than the other patients (OR: 1 vs 0.215 [95% CI: 0.137–0.388]). HER2 positive with H treated patients (P < 0.001) but not those without H treated (P = 0.361) had statistically significant higher probability of ALN pCR rate than HER2 negative patients (OR: 6.004 [95% CI: 3.256–11.072] vs 1). The axillary pCR rate of patients with high levels of Ki67 expression (Ki67 > 20%) was higher than that of patients with low levels of Ki67 expression (P < 0.001, OR = 2.627 [95% CI: 1.651–4.179] vs 1). Patients received PC regimen had statistically significant higher ALN pCR rate than those received E + P (P = 0.003, OR = 2.220 [95% CI: 1.320–3.735] vs 1). No significant difference in the axillary pCR rate was observed among patients with different NCT cycles (P = 0.341). Univariate analysis of the training set and the validation set showed similar results compared with patients in the total population.

### Predictors for ALN pCR

Multivariate logistic regression analysis was performed to identify independent variables for predicting the pCR of the ALN post-NCT (Table 2). Predictors that were statistically significant (P < 0.05) in the univariate logistic analysis (tumor size, Ki67, HR status, HER2 status and NCT regimens) were included in the multivariate logistic regression analysis. According to the results, HR positive patients (P < 0.001, OR = 0.162 [95% CI: 0.074–0.353]) were less likely to show ALN pCR than HR negative patients. In contrast, HER2 positive patients received H (P = 0.024, OR = 3.443 [95% CI: 1.178–10.060]) were more likely to show ALN pCR than HER2 negative patients, and patients with Ki67 > 20% (P = 0.037, OR = 2.258 [95% CI: 1.049–4.862]) were more likely to show ALN pCR than those with Ki67 ≤ 20%. Tumor size and NCT regimens were not significantly associated with ALN pCR.

### Construction and Validation of the Nomogram

**Figure 2:**
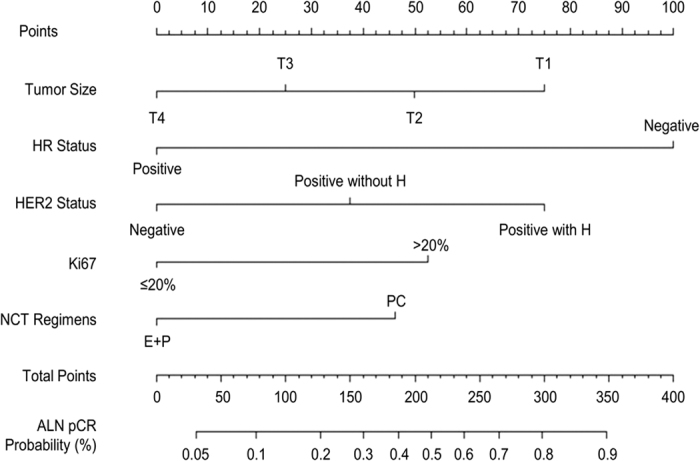
A nomogram predicting the probability of pathological complete response (pCR) of axillary lymph nodes (ALNs) after neoadjuvant chemotherapy (NCT). E + P, epirubicin + paclitaxel contained; H, Herceptin; HER2, human epithelial growth factor receptor 2; HR, hormone receptor; PC, paclitaxel and carboplatin or paclitaxel and cisplatin.

Independent predictors identified in the multivariate logistic regression analysis (P < 0.05), including HR status, HER2 status and Ki67 index as well as tumor size and NCT regimens were utilized to construct the nomogram. The total points were summed up by the points of each variable (top plotting scale). The ALN pCR probability was subject to the total points (bottom plotting scale). The P-value for the Hosmer and Lemeshow test was 0.626, indicting a good fit to the model. The final nomogram is shown in Fig. 2.

**Figure 3:**
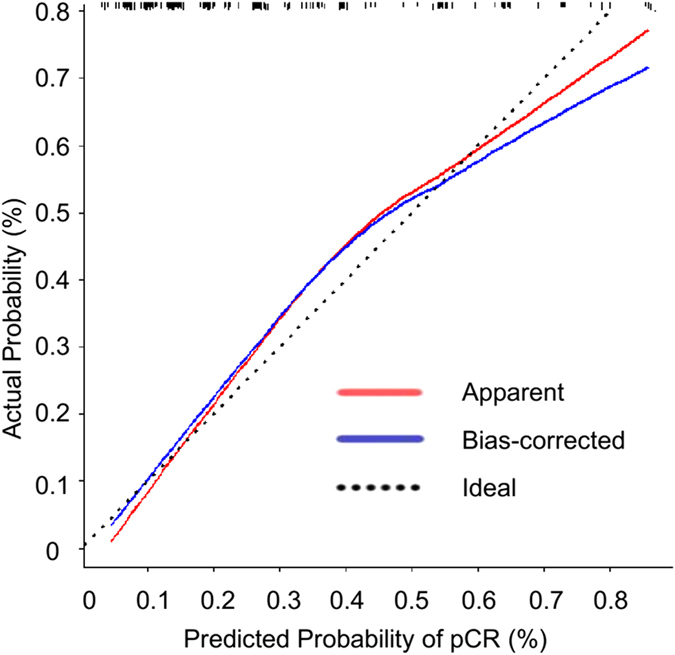
Calibration plot of the nomogram for the probability of pathological complete response (pCR) of axillary lymph node (ALN) (bootstrap 1000 repetitions).

The calibration of the nomogram was performed internally by a calibration plot with bootstrap sampling (n = 1000) (Fig. 3). Calibration plot of an accurate model may fall along the 45-degree line. Our bias-corrected curve was close to the ideal curve, which indicated that the nomogram was well calibrated.

**Figure 4:**
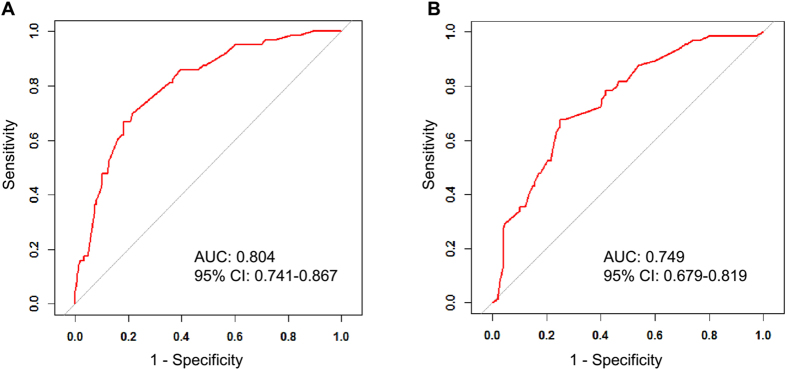
Validation of the nomogram. (**A**) Internal validation using the receiver operating characteristic (ROC) curve. The area under the ROC curve (AUC) is 0.804, 95% confidence interval (95% CI, 0.741–0.867). (**B**) External validation using ROC. The AUC is 0.749 (95% CI, 0.679–0.819).

Next, the ROC was performed to validate the nomogram internally in the training set (Fig. 4A) and externally in the validation set (Fig. 4B). In the training set, the AUC was 0.804 (95% CI: 0.741–0.867). In the validation set. The AUC was 0.749 (95% CI: 0.679–0.819). The difference between two AUCs was not statistical significant (P = 0.253). These results illustrated that the predicted and observed ALN pCR probabilities were in good concordance, and the goodness of fit of the nomogram was favorable.

Values of sensitivity, specificity, and predictive values of the predicted probability at different cutoff values of the nomogram were shown in Supplementary material 2. Higher cutoff value resulted in the increasing of specificity and positive predictive value, sensitivity and negative predictive value decreased. The diagnostic odds ratios of the nomogram at different cutoff values were shown in Supplementary material 3. The cutoff values for the good performance for the nomogram ranged between ≥0.2 to ≥0.6 in the training set and ≥0.2 to ≥0.4 in the validation set. At last, the optimal cutoff values of the training and validation sets were 0.34 (sensitivity: 66.7%, specificity: 82.0%, positive predictive value: 60.9%, negative predictive value: 85.4%) and 0.27 (sensitivity: 67.7%, specificity: 75.0%, positive predictive value: 54.3%, negative predictive value: 84.1%), respectively (Supplementary Material 4).

### The Application of the Nomogram

To display the application of the nomogram, we took two pathologically proven node positive breast cancer patients who had received NCT as examples. The first patient’s tumor size was T1 (75 points). Her HR status was negative (100 points), and her HER2 status was positive and she received H for treatment (75 points). Her Ki67 index was more than 20% (53 points). Her NCT regimen was PC (46 points). According to her total points (349 points), she had a high probability (>0.9) of showing ALN pCR post-NCT, and her actual ALN status after NCT was pCR. The second patient’s tumor size was T4 (0 points). Her HR status was negative (100 points), and her HER2 status was negative (0 points). Her Ki67 index was no more than 20% (0 points). Her NCT regimen was E + P (0 points). According to her total points (100 points), she had a relatively low probability of reaching ALN pCR post-NCT (0.1–0.2), and her actual ALN status after NCT was not pCR.

## Discussion

In our current research, we first randomized the population containing 426 pathologically proven ALN positive patients who received NCT into a training set and a validation set. Based on the multivariate logistic regression analysis, we identified independent variables for predicting the ALN pCR after NCT. Patients who were HR positive were less likely to show ALN pCR than those who were HR negative. Patients who were HER2 positive and reveived H were more likely to show ALN pCR than those who were HER2 negative, and patients with Ki67 > 20% were more likely to show ALN pCR than those with Ki67 ≤ 20%. Tumor size and NCT regimens did not showed statistically significance in the multivariate logistic regression analysis. Next, we constructed a nomogram on the basis of these predictors as well as clinical significant predictors (tumor size and NCT regimens). The AUCs of the ROCs in the training (internal validation) and validation sets (external validation) were 0.804 and 0.749, respectively.

At present because of the lack of reliable diagnostic approaches to evaluate lymph node status after receiving NCT for pathologically proven node positive breast cancer patients^21^, the standard treatment for them is mastectomy together with ALN dissection. However, several previous studies concluded that the ALN dissection might cause physical complications, including numbness, weakness, pain, limb swelling and stiffness as well as psychological disorders^4^,^22^,^23^,^24^,^25^,^26^. Consequently, it is of great importance to improve the accuracy of SLNB post-NCT and to avoid unnecessary ALN dissection.

According to our results, patients who were HR negative, HER2 positive and reveived H or had higher Ki67 expression were more likely to show ALN pCR than the other patients. Previous studies showed high concordance rates of receptor status (ER, PR and HER2) between primary and ALN metastatic lesions^27^,^28^. Because we could not assess the expression of HR, HER2 or Ki67 in the ALN, we presumed that the expression of HR, HER2 and Ki67 in the primary tumor could be substituted for that in the ALN metastasis. The HR negative and HER2 positive patients had higher ALN pCR rates because HR negative and HER2 positive tumor cells are more sensitive to NCT^29^,^30^,^31^,^32^. Alvarado *et al*. reported that HER2 positive patients had a higher rate of ALN pCR than HER2 negative patients, which was consistent with our data^33^. Schipper *et al*. enrolled 291 patients with clinically proven node positive breast cancer^34^. Similar to our study, their results showed that HR negative patients and HER2 positive patients were more likely to show ALN pCR than other patients. However, they did not perform the multivariate logistic regression analysis to identify independent variables for predicting the ALN pCR post-NCT. Ki67 is a proliferation marker, and it provides a quick method to evaluate the proportion of proliferating cells within a tumor (higher Ki67 levels indicate more proliferating cells)^35^. Previous research showed that patients with higher Ki67 levels showed higher frequencies of pCR^36^, which was consistent with our current research.

The nomogram, a simple graphical prediction tool, allows oncologists to assess the predictive risk of individuals^37^. Another advantage of the nomogram that it is noninvasive. One previous study constructed a nomogram to predict ALN pCR among clinically proven node positive patients post-NCT^34^. The defect of that study was that it did not screen the predictors and perform the multivariate logistic regression analysis to identify independent variables for predicting the ALN pCR post-NCT. Moreover, the investigators did not perform an external validation of the model. The nomogram we constructed is concise (5 predictors) and powerful for predicting ALN pCR. It thus increases the accuracy of SLNB post-NC T and helps to assess the actual ALN status post-NCT. ACOSOG Z1071 trial reported that placing clips in suspicious nodes before NCT could decrease the FNR of SNB after NCT^9^. But clips of only half patients could be found in surgery so this technique was not recommended^9^. Previous investigators proposed that applying a dual agent mapping method together with removing more than 3 SLNs might minimize the FNR of SLNB post-NCT^9^,^11^,^12^.

Recently, You *et al*. evaluated the diagnostic value of ultrasound, MRI and PET/CT for ALN metastases after NCT^38^. Combination of all three imaging tests showed highest sensitivity for the detection of positive ALN metastases.

Combined with our nomogram, the accuracy of SLNB post-NCT can be further improved. According to our nomogram, patients with high points were more likely to show ALN pCR. If their SLNB post-NCT showed no metastases in the lymph nodes, they could safely avoid receiving ALN dissection.

One major limitation of our study was that the design was a single-center retrospective analysis and we did not compare the predictive power of SLNB together with the nomogram with the SLNB alone because of the lack of data. In the future, two related randomized prospective clinical trials in progress may help to validate the nomogram. The first one is NCT01901094, which evaluates the efficacy of ALN dissection compared with ALN radiation therapy in ALN positive breast cancer patients convert to negative after NCT. The second one is NSABP B-51 (NCT01872975), which evaluates whether comprehensive radiation therapy in ALN after surgery may improve prognosis in ALN positive breast cancer patients convert to negative after NCT. Those researches will also investigate the survival benefits of avoiding ALN dissection for some patients.

## Conclusion

We constructed a nomogram for predicting post-NCT ALN pCR. With the nomogram, we can predict post-NCT ALN status accurately and avoid unnecessary ALN dissection.

## Additional Information

**How to cite this article**: Jin, X. *et al*. A Nomogram for Predicting the Pathological Response of Axillary Lymph Node Metastasis in Breast Cancer Patients. *Sci. Rep.* **6**, 32585; doi: 10.1038/srep32585 (2016).

## Supplementary Material

Supplementary Information

## Figures and Tables

**Table 1:** Clinicopathological characteristics and univariate logistic regression analysis of different variables predicting ALN pCR of the total population, the training set and the validatio set.

	Overall population					Trainning set				Validation set					
	Overall (No.)	Axillary pCR (No.)	Axillary pCR rate	P	OR (95% CIs)	Overall (No.)	Axillary pCR (No.)	Axillary pCR rate	P	OR (95% CIs)	Overall (No.)	Axillary pCR (No.)	Axillary pCR rate	P	OR (95% CIs)
Total	426	128	30.0%			213	63	29.6%			213	65	30.5%		
Age				0.897					0.416					0.536	
≤40 years	75	23	30.7%		1	37	13	35.1%		1	38	10	26.3%		1
>40 years	351	105	29.9%	0.897	0.965 (0.562–1.658)	176	50	28.4%	0.416	0.733 (0.346–1.551)	175	55	31.4%	0.536	1.283 (0.583–2.826)
Menopausal Status				0.849					0.098					0.054	
pre-menopausal	216	64	29.6%		1	103	36	35.0%		1	113	28	24.8%		1
post-menopausal	210	64	30.5%	0.849	1.041 (0.688–1.575)	110	27	24.5%	0.098	0.605 (0.334–1.096)	100	37	37.0%	0.054	1.783 (0.989–3.214)
Tumor Size				0.002					0.029					0.035	
T1	37	20	54.1%		1	15	7	46.7%		1	22	13	59.1%		1
T2	219	69	31.5%	0.009	0.391 (0.193–0.793)	113	40	35.4%	0.398	0.626 (0.212–1.854)	106	29	27.4%	0.006	0.261 (0.101–0.675)
T3	75	14	18.7%	<0.001	0.195 (0.082–0.465)	43	6	14.0%	0.013	0.185 (0.049–0.702)	32	8	25.0%	0.014	0.231 (0.072–0.742)
T4	95	25	26.3%	0.003	0.304 (0.138–0.670)	42	10	23.8%	0.103	0.357 (0.104–1.232)	53	15	28.3%	0.014	0.273 (0.097–0.772)
HR Status				<0.001					<0.001					<0.001	
Negative	124	67	54.0%		1	58	34	58.6%		1	66	33	50.0%		1
Positive	302	61	20.2%	<0.001	0.215 (0.137–0.388)	155	29	18.7%	<0.001	0.162 (0.084–0.314)	147	32	21.8%	<0.001	0.278 (0.149–0.518)
HER2 Status				<0.001					<0.001					<0.001	
Negative	321	77	24.0%		1	158	36	22.8%		1	163	41	25.2%		1
Positive without H	50	15	30.0%	0.361	1.358 (0.704–2.619)	30	10	33.3%	0.221	1.694 (0.728–3.946)	20	5	25.0%	0.988	0.992 (0.339–2.898)
Positive with H	55	36	65.5%	<0.001	6.004 (3.256–11.072)	25	17	68.0%	<0.001	7.201 (2.873–18.050)	30	19	63.3%	<0.001	5.14 (2.258–11.699)
Ki67				<0.001					0.005					0.003	
≤20%	167	31	18.6%		1	82	15	18.3%		1	85	16	18.8%		1
>20%	259	97	37.5%	<0.001	2.627 (1.651–4.179)	131	48	36.6%	0.005	2.583 (1.331–5.014)	128	49	38.3%	0.003	2.675 (1.396–5.125)
NCT Regimens				0.003					0.01					0.067	
E + P	82	21	25.6%		1	53	8	15.1%		1	61	13	21.3%		1
PC	310	106	34.2%	0.003	2.220 (1.320–3.735)	160	55	34.4%	0.01	2.946 (1.298–6.688)	152	52	34.2%	0.067	1.92 (0.955–3.860)
NCT Cycles				0.341					0.829					0.271	
1–4	316	91	28.8%		1	161	47	29.2%		1	155	44	28.4%		1
5–8	110	37	33.6%	0.341	1.253 (0.788–1.994)	52	16	30.8%	0.829	1.078 (0.546–2.128)	58	21	36.2%	0.271	1.432 (0.756–2.713)

Abbreviations: ALN, axillary lymph node; CI, confidence interval; E + P, epirubicin + paclitaxel contained; H, Herceptin; HR, hormone receptor; HER2, human epithelial growth factor receptor 2; NCT, neoadjuvant chemotherapy; OR, odds ratio; PC, paclitaxel and carboplatin or paclitaxel and cisplatin; pCR, pathological complete response.

**Table 2:** Multivariate logistic regression analysis of variables (P < 0.05 in univariate logistic regression analysis) predicting ALN pCR.

Predictor	P	OR (95% CIs)
Tumor Size		
T1		1
T2	0.754	1.257 (0.300–5.364)
T3	0.104	0.25 (0.047–1.330)
T4	0.577	0.637 (0.130–3.111)
HR Status		
HR negative		1
HR positive	<0.001	0.162 (0.074–0.353)
HER2 Status		
HER2 negative		1
HER2 positive without H	0.185	1.876 (0.739–4.758)
HER2 positive with H	0.024	3.443 (1.178–10.060)
Ki67		
≤20		1
>20	0.037	2.258 (1.049–4.862)
NCT Regimens		
E + P		1
PC	0.177	1.899 (0.749–4.811)

Abbreviations: ALN, axillary lymph node; CI, confidence interval; E + P, epirubicin + paclitaxel contained; H, Herceptin; HR, hormone receptor; HER2, human epithelial growth factor receptor 2; NCT, neoadjuvant chemotherapy; OR, odds ratio; PC, paclitaxel and carboplatin or paclitaxel and cisplatin; pCR, pathological complete response.
