# Supplementary material for: A Nomogram for Predicting the Pathological Response of Axillary Lymph Node Metastasis in Breast Cancer Patients
Source: Sci Rep. 2016 Aug 31;6:32585. doi: 10.1038/srep32585 (PMC5006169; doi:10.1038/srep32585)
Supplement: Supplementary Information [file srep32585-s1.pdf]

A Nomogram for Predicting the Pathological Response of Axillary Lymph Node Metastasis in Breast Cancer Patients

Xi Jin[1#], Yi-Zhou Jiang1[*#], Sheng Chen[1], Zhi-Ming Shao[1], Gen-Hong Di[1*]

1 Department of Breast Surgery, Fudan University Shanghai Cancer Center; Department of Oncology, Shanghai Medical College, Fudan University, Shanghai 200032, China

# These authors contributed equally to this work.

*Corresponding author:

Gen-Hong Di

Email: genhongdi@163.com

Work telephone number: +86-64175590

Fax: +86-21-64434556

Yi-Zhou Jiang

Email: yizhoujiang@fudan.edu.cn

Work telephone number: +86-64175590

Fax: +86-21-64434556

Running title: Nomogram for predicting ALN pCR

Supplementary material 1: R running of the nomogram for predicting axillary lymph nodes pathologically complete response. This is the code of nomogram development and validation using R software.

##Nomogram Construction

```
library(rms)
F1<-read.csv(file.choose(),header=T)
attach(F1)
ddist<-datadist(T,HR,HER2,ALNpCR,Ki67,Age,Meno,Regimens)
options(datadist='ddist')
logi<-lrm(ALNpCR~T+HR+HER2+Ki67+Regimens,x=TRUE, y=TRUE)
nomo<-nomogram(logi, fun=plogis, fun.at=c(.001, .01, .05, seq(.1,.9, by=.1), .95, .99, .999),lp=F, funlabel="ALNpCR")
plot(nomo)
```

Predictors		Points
Tumor Size	T1	75
	T2	50
	T3	25
	T4	0
HR Status	Negative	100
	Positive	0
HER2 Status	Negative	0
	Positive without H	38
	Positive with H	75
Ki67	≤20%	0
	>20%	53
NCT Regimens	E+P	0
	PC	46

Total Points	ALN pCR Rate
31	0.05
77	0.1
127	0.2
160	0.3
188	0.4
213	0.5
238	0.6
265	0.7
298	0.8
348	0.9

##Calibrate curve construction

```
cal<-calibrate(logi, method="boot", B=1000, bw=FALSE, rule="p", type="individual", sls=.05, aics=0, force=NULL, estimates=T, pr=FALSE, smoother="lowess", digits=NULL)
plot(cal,scat1d.opts=list(nhistSpike=500))
```

## ROC of Training set construction

```
library(pROC)
F1<-read.csv(file.choose(),header=T)
attach(F1)
logi<-lrm(ALNpCR~T+HR+HER2+Ki67+Regimens,x=TRUE, y=TRUE)
Predi<-predict(logi,F1,type="lp")
roc1<-roc(F1$ALNpCR,Predi,legacy.axes=TRUE)
plot(roc1,font=2,legacy.axes=TRUE)
Area under the curve: 0.8038
ci.auc(roc1)
95% CI: 0.7411-0.8666 (DeLong)
```

## ROC of Validation set construction

```
F2<-read.csv(file.choose(),header=T)
Predi<-predict(logi,F2,type="lp")
roc2<-roc(F2$ALNpCR,Predi,legacy.axes=TRUE)
plot(roc2,font=2,legacy.axes=TRUE)
Area under the curve: 0.7491
ci.auc(roc2)
95% CI: 0.6794-0.8187 (DeLong)
```

## Compare the AUC of two ROC curves

```
roc.test(roc1,roc2)
DeLong's test for two ROC curves
data:   jxroc1 and jxroc2
D = 1.5077, df = 650.34, p-value = 0.1321
alternative hypothesis: true difference in AUC is not equal to 0
sample estimates:
AUC of roc1 AUC of roc2
   0.7793607     0.7029754
```

Supplementary material 2: Values of sensitivity, specificity, and predictive values of the predicted probability at different cutoff values.

Predicted Probability	Training Set				Validation Set			
	Sensitivity	Specificity	PPV	NPV	Sensitivity	Specificity	PPV	NPV

≥0.1	96.8%	26.0%	35.5%	95.1%	95.4%	29.1%	37.1%	93.5%
≥0.2	85.7%	60.7%	47.8%	91.0%	78.5%	57.4%	44.7%	85.9%
≥0.3	66.7%	79.3%	57.5%	85.0%	64.6%	75.0%	53.2%	82.8%
≥0.4	52.4%	87.3%	63.5%	81.4%	52.3%	79.7%	53.1%	79.2%
≥0.5	47.6%	89.3%	65.2%	80.2%	43.1%	84.5%	54.9%	77.2%
≥0.6	36.5%	92.7%	67.6%	77.7%	35.4%	89.2%	59.0%	75.9%
≥0.7	17.5%	96.7%	68.8%	73.6%	27.7%	95.9%	75.0%	75.1%
≥0.8	11.1%	99.3%	87.5%	72.7%	3.1%	98.0%	40.0%	69.7%

NPV: negative predictive value; PPV: positive predictive value.

Supplementary material 3

The diagnostic odds ratio (DOR) of the nomogram in the training set (A) and the validation set (B) at different cutoff values. CI: confidence interval.

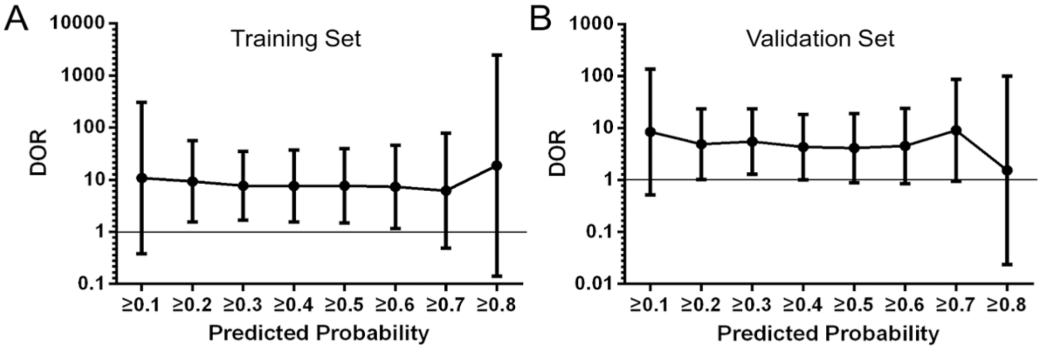

DOR and 95% CI at Different Cutoff Values

Predicted Probability	DOR (95% CI)
≥0.1	10.716 (0.376-305.488)
≥0.2	9.254 (1.543-55.508)
≥0.3	7.677 (1.694-34.787)
≥0.4	7.584 (1.549-37.138)
≥0.5	7.614 (1.463-39.627)
≥0.6	7.266 (1.152-45.841)
≥0.7	6.135 (0.483-77.873)
≥0.8	18.625 (0.142-2442.456)

DOR and 95% CI at Different Cutoff Values

Predicted Probability	DOR (95% CI)
≥0.1	8.463 (0.520-137.827)
≥0.2	4.915 (1.038-23.266)
≥0.3	5.478 (1.284-23.370)
≥0.4	4.314 (1.010-18.423)
≥0.5	4.113 (0.895-18.904)
≥0.6	4.518 (0.848-24.064)
≥0.7	9.064 (0.947-86.770)
≥0.8	1.534 (0.024-99.872)

Supplementary material 4: Values of sensitivity, specificity, and predictive values of the predicted probability at the optimal cutoff value.

	The Optimal Cutoff*	Sensitivity	Specificity	PPV	NPV
Training Set	0.34	66.7%	82.0%	60.9%	85.4%
Validation Set	0.27	67.7%	75.0%	54.3%	84.1%

NPV: negative predictive value; PPV: positive predictive value.

* The optimal cutoff value is determined according to Youden's method[1].

REFERENCES

1 Youden W.J., Index for rating diagnostic tests. *Cancer* **3**, 32-35 (1950).
